# Supplementary figures and images for: Polypharmacology-based kinome screen identifies new regulators of KSHV reactivation
Source: PLoS Pathog. 2023 Sep 5;19(9):e1011169. doi: 10.1371/journal.ppat.1011169 (PMC10503724; doi:10.1371/journal.ppat.1011169)

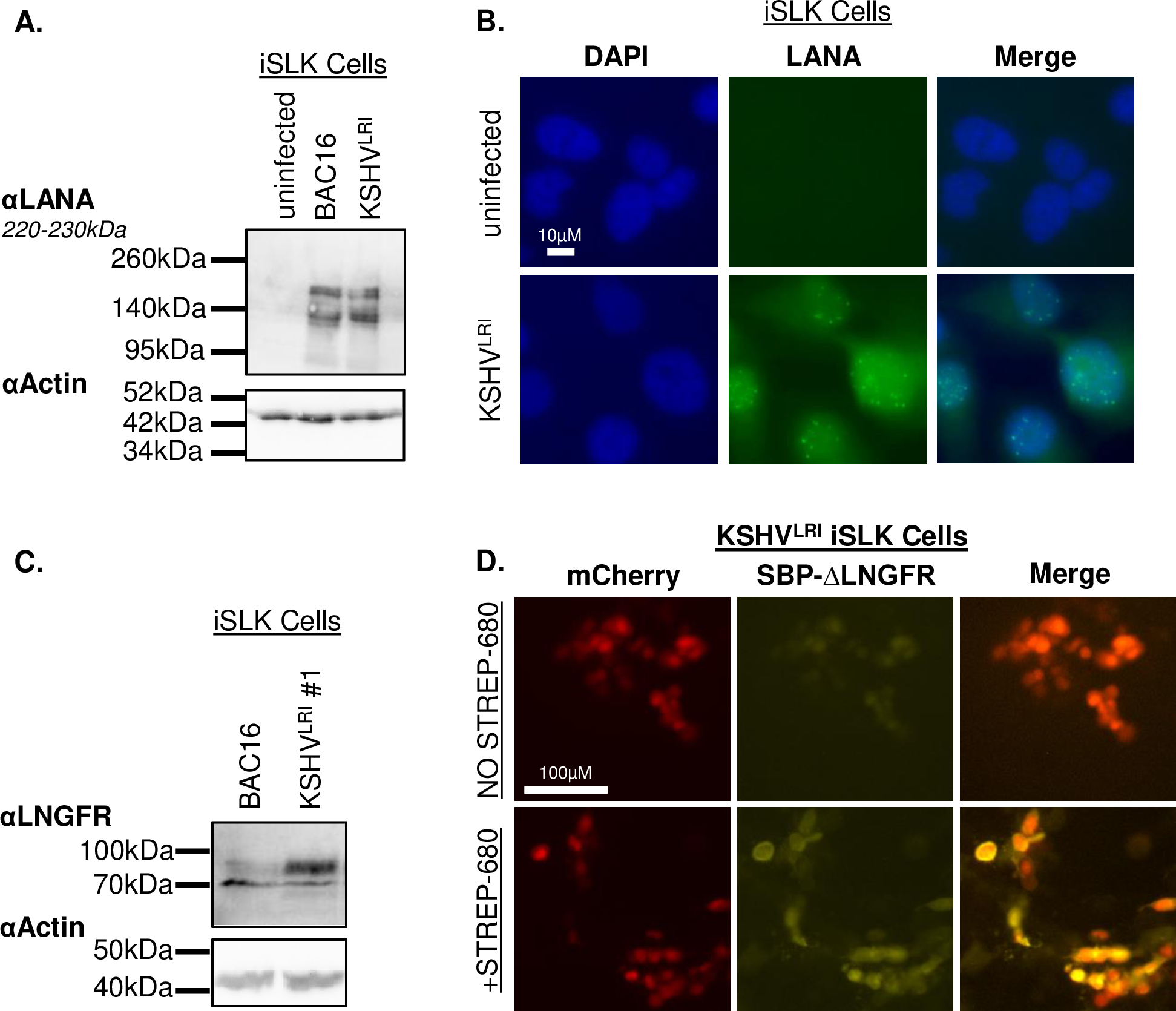

Supplement: S1 Fig — Uninfected iSLK cells or KSHV BAC16 or KSHVLRI latently infected iSLK cells were (A) lysed and subjected to LANA immunoblotting or (B) analyzed by immunofluorescence for LANA puncta representing individual KSHV episomes. (C) KSHV BAC16 or KSHVLRI latently infected iSLK cells were treated with 1 μg/ml DOX plus 1 mM NaB and incubated for 3-days before harvesting cells for immunoblot analysis of SBP-ΔLNGFR protein levels or (D) fixed and incubated with streptavidin-680 for imaging of SBP-ΔLNGFR on the plasma membrane of un-permeabilized cells. (TIF) [file ppat.1011169.s001.tif]

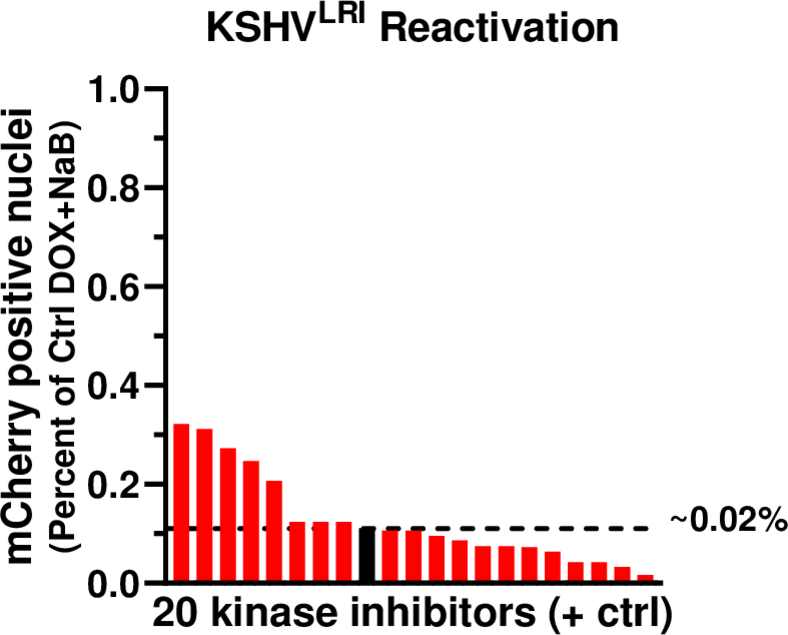

Supplement: S2 Fig — KSHVLRI reactivation phenotypes were obtained from 20 of the 29 pre-selected KIs that had minimal changes (< 30%) to cell confluences as compared to the DMSO control as a measurement of cell viability and that demonstrated consistent dose responses curves. KSHV reactivation for control (black bar and dotted black line) and KI treatment (red bars) were calculated as a percent of DOX plus NaB treated cells set to 100 from data in Fig 2C. In this graph, 1.0 represents ~0.2% of total cells and the dotted line represents spontaneous reactivation, ~0.02% of total cells or ~2 mCherry positive cells. (TIF) [file ppat.1011169.s002.tif]

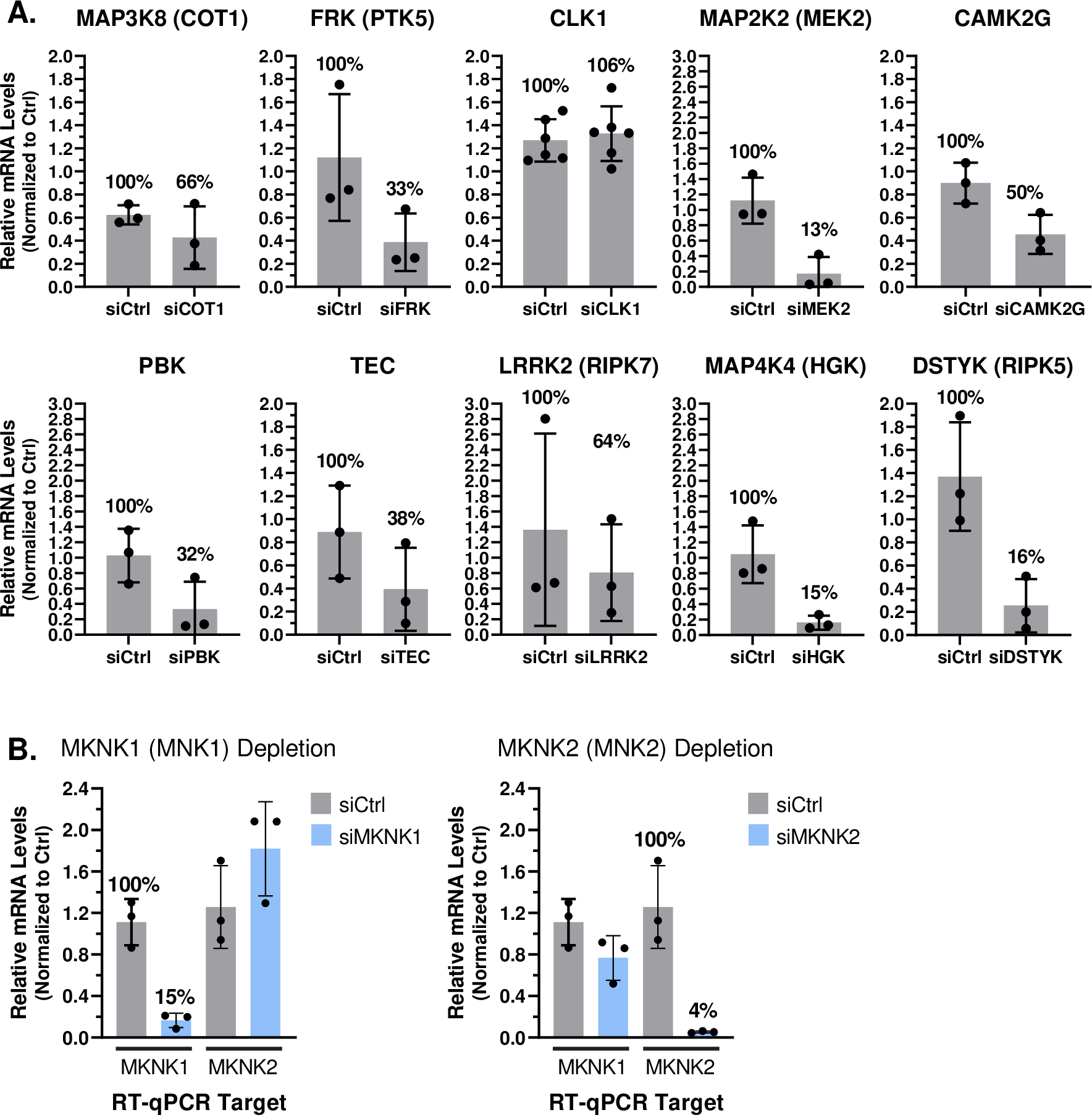

Supplement: S3 Fig — Knockdown efficiencies for siRNAs targeting specific cellular kinases were evaluated in KSHVLRI latently infected iSLK cells using RT-qPCR from total RNA harvested at 3-days post transfection with siRNAs. Relative mRNA levels were normalized to untransfected control cells (Ctrl) by setting this to 100 on the y-axis. The siCtrl transfected cells (siCtrl) were used to calculate the relative mRNA levels in the kinase-specific siRNA treated cells which are listed above each bar in the graphs. Data for ERBB1-4 are in S4 Fig. (TIF) [file ppat.1011169.s003.tif]

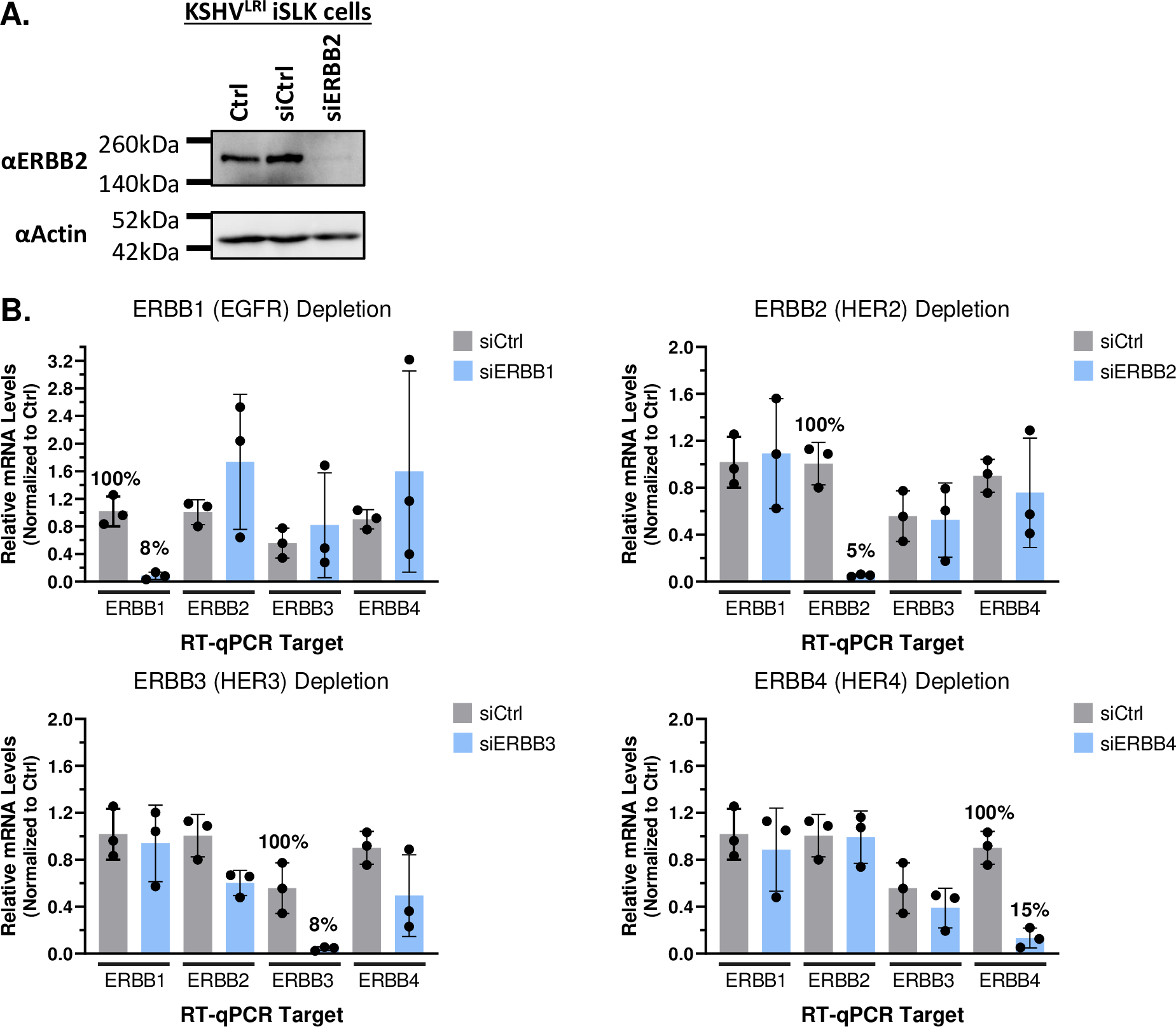

Supplement: S4 Fig — (A) Knockdown efficiency of ERBB2 targeting siRNA was evaluated by immunoblot for ERBB2 protein at 2 days following siRNA transfection of KSHVLRI latently infected iSLK cells. (B) Knockdown specificity for siRNAs targeting ERBB family kinases were evaluated in KSHVLRI latently infected iSLK cells using RT-qPCR from total RNA harvested at 3-days post transfection with siRNAs. Relative mRNA levels were normalized to untransfected control cells (Ctrl) by setting this to 100 on the y-axis. The siCtrl transfected cells (siCtrl) were used to calculate the relative mRNA levels in the kinase specific siRNA treated cells which are listed above each bar in the graphs. (TIF) [file ppat.1011169.s004.tif]

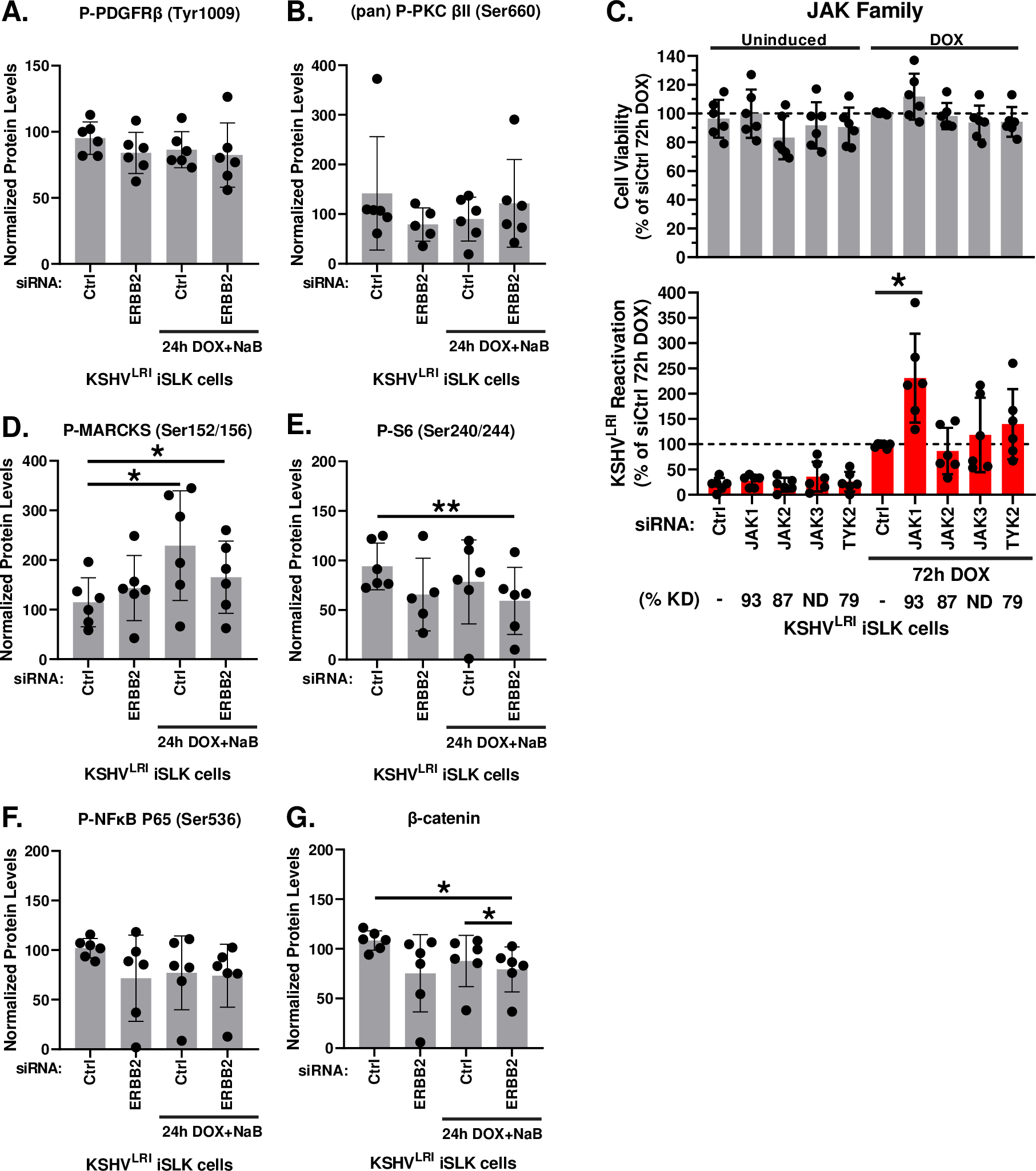

Supplement: S5 Fig — KSHVLRI latently infected iSLK cells were transfected with siRNA control or siRNAs targeting ERBB2 and then 3-days later untreated or treated with DOX plus NaB for 24h. Cells were harvested, and protein lysates were analyzed using a RPPA for phosphorylation of (A) plasma membrane receptor PDGFRβ at Tyr1009 and (B) signaling intermediates (pan) PKC at Ser660. Relative phospho-protein levels under each condition were normalized to untransfected control cells (Ctrl) by setting this to 100 on the y-axis. (C) Cell viability (grey bars) and KSHV reactivation (red bars) were measured for KSHVLRI latently infected iSLK cells transfected with siRNAs targeting individual JAK family kinases and 3-days later uninduced or treated with DOX alone for 72h. Control siRNA transfected cells treated with DOX (dotted black lines) were set to 100 and data for each condition was calculated as a percent of this control. Kinase knockdown efficiencies at 3-days following siRNA transfection were determined before addition of lytic inducing drugs and graphed in S6 Fig. For each knockdown, the efficiencies were averaged and listed below the corresponding kinase target as % KD. Identical to (A and B), quantification of phosphorylated (D) MARKS at Ser152/156, (E) S6 at Ser240/244, (F) NFκB P65 at Ser536, and (G) total β-catenin protein were analyzed. Paired for (A,B,D-G) or unpaired (C) t tests were performed in Excel for each kinase knockdown condition compared to siCtrl or siCtrl with 24h DOX+NaB or for KI as compared to DMSO control.P-values * ≤ 0.05 and ** ≤ 0.01. (TIF) [file ppat.1011169.s005.tif]

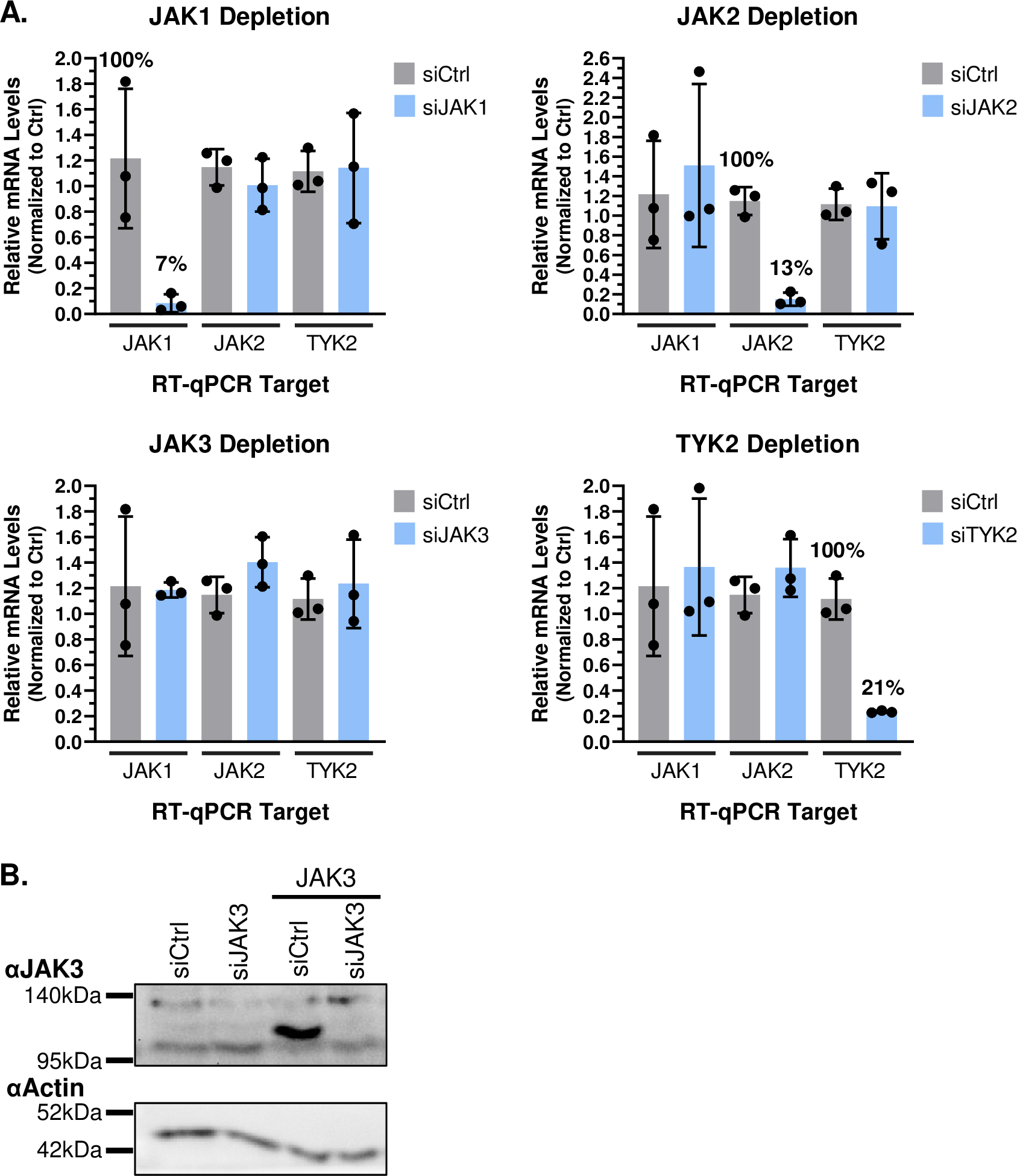

Supplement: S6 Fig — (A) Knockdown specificity for siRNAs targeting JAK1, JAK2, JAK3 and TYK2 were evaluated using RT-qPCR from total RNA harvested at 3-days post transfection with siRNAs. Relative mRNA levels for JAK3 were below the level of detection for these samples. (B) HeLa cells were transfected with control or JAK3 targeting siRNA alone or in combination with a JAK3 expressing plasmid. Three days post transfection cells were harvested, and lysates were subjected to α-JAK3 and α-Actin immunoblotting. (TIF) [file ppat.1011169.s006.tif]

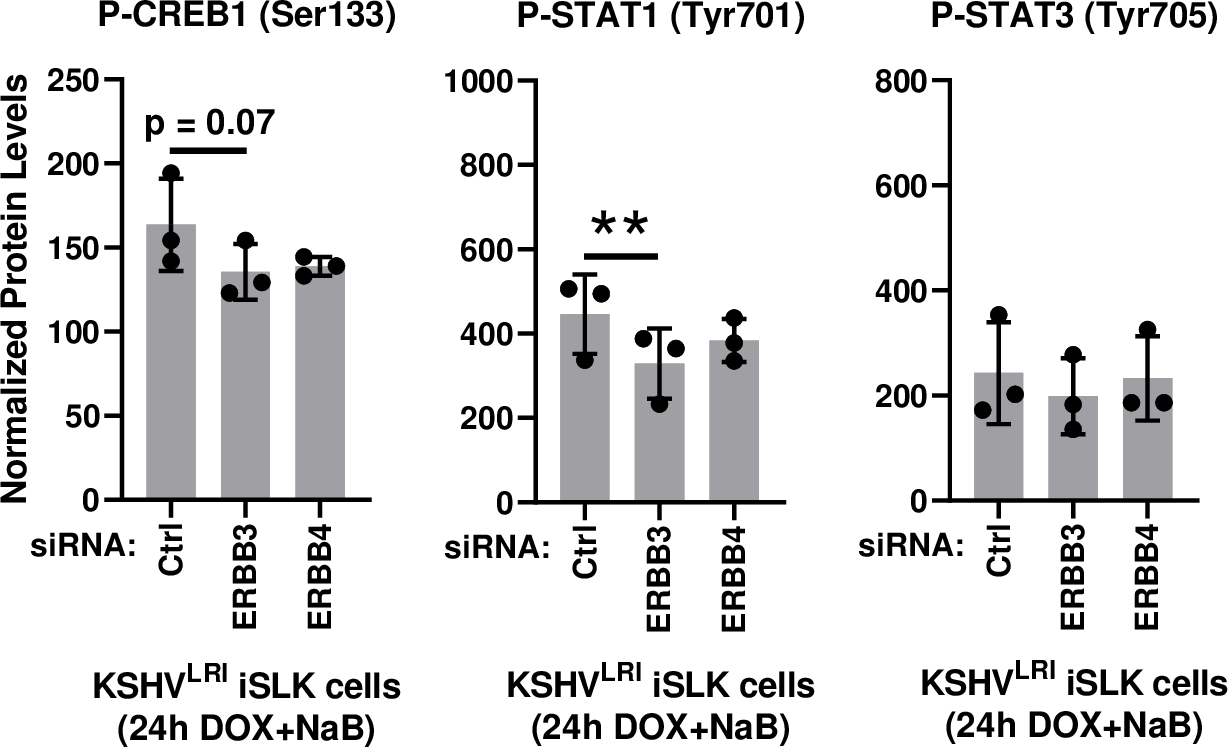

Supplement: S7 Fig — KSHVLRI latently infected iSLK cells were transfected with siRNA control or siRNAs targeting ERBB2, ERBB3 or ERBB4 and then 3-days later untreated or treated with DOX plus NaB for 24h. Cells were harvested, and protein lysates were analyzed using a RPPA for phosphorylation of CREB1 at Ser133, STAT1 at Tyr701, and STAT3 at Tyr705. Relative phospho-protein levels were normalized to untransfected control cells (Ctrl) by setting this to 100 on the y-axis. Paired t tests were performed in Excel for each kinase knockdown condition compared to siCtrl or siCtrl with 24h DOX+NaB. P-values * ≤ 0.05, ** ≤ 0.01, and *** ≤ 0.001. (TIF) [file ppat.1011169.s007.tif]
