# Supplementary material for: Polypharmacology-based kinome screen identifies new regulators of KSHV reactivation
Source: PLoS Pathog. 2023 Sep 5;19(9):e1011169. doi: 10.1371/journal.ppat.1011169 (PMC10503724; doi:10.1371/journal.ppat.1011169)
Supplement: Attachment — Submitted filename: PLospathogens-kinome review.docx [file ppat.1011169.s019.docx]

In this manuscript, Olson et al., utilized polypharmacology-based screening of kinase inhibitors to identify new modulators of KSHV lytic reactivation. For their screening, they generated a stably-infected iSLK cells with new KSHV recombinant virus that contains infection and lytic replication indicators. They then performed the kinase inhibitors screening with the newly-generated iSLK stable cells on 29 compounds and used this initial screening results as the training set for the kinome regularization pipeline (in silico screening) to determine KSHV lytic regulators. Overall, the idea underlying this work is novel and interesting. The whole pipeline has potential applicability in other latent infections as well. ERBB’s role in KSHV lytic is novel and exciting finding. The manuscript is also well written. There are only a few minor revision points to improve the overall manuscript as follow:

1. In this paper, the authors used selection of 29 compounds for their initial screening, which target around half of the human kinome. They should briefly describe the reasoning or the computational set up for this selection.
2. The authors should also address the suitability of these 29 inhibitors as a training set since they are not selective (collectively targeting 296 kinases).
3. How selective are the top inhibitors in targeting each of the 13 kinases vs other non-kinase cellular functions? It seems that around 50% of the kinases listed in table s3 are validated
4. The authors put forward this model of ERBB2 switching interaction partners from ERBB1 during latency and ERBB3 in lytic, but there is no direct evidence showing of these heterodimerizations and switching.
5. The paper should also show interference of ERBB2 heterodimerizations leads to lytic reactivation or lytic inhibition (ie. Latency) to further support the model presented in the manuscript.
6. How do the authors ascertain ERBB2:3 vs ERBB2:4 induces CREB1 phosphorylation and lytic reactivation (Fig 9) since ERBB4 is one of the strongest pro-lytic factors found from their screening and validation (Fig 3)? Exogenous expression of ERBBs or other more appropriate cell lines can be used if endogenous expression is low.
7. Does ligand binding affect the heterodimerization switching? Or if any other conditions induce the switching? It would be nice to link it to KSHV pathogenesis.
