# Supplementary material for: Polypharmacology-based kinome screen identifies new regulators of KSHV reactivation
Source: PLoS Pathog. 2023 Sep 5;19(9):e1011169. doi: 10.1371/journal.ppat.1011169 (PMC10503724; doi:10.1371/journal.ppat.1011169)
Supplement: Attachment — Submitted filename: rebuttal letter w refs 7.6.23.docx [file ppat.1011169.s020.docx]

In the revised manuscript we have addressed the issues raised by the Editors and Reviewers by performing several additional experiments and by editing the text and figures to be clearer and more precise. While we are not able to answer all the issues raised by the reviewers definitively, in part because signaling pathways are very complex, we think our report demonstrates the successful application of a novel and widely applicable screening technology, resulting in the identification of cellular kinases with previously unappreciated roles in the KSHV life cycle. Thus, we think this work represents an important advance that will be of considerable interest to the readers of PLoS Pathogens.

**Letter from Editor.** In this manuscript, Olson et al., utilized polypharmacology-based screening of kinase inhibitors to identify new modulators of KSHV lytic reactivation. For their screening, they generated a stably-infected iSLK cells with new KSHV recombinant virus that contains infection and lytic replication indicators. They then performed the kinase inhibitors screening with the newly-generated iSLK stable cells on 29 compounds and used this initial screening results as the training set for the kinome regularization pipeline (in silico screening) to determine KSHV lytic regulators. Overall, the idea underlying this work is novel and interesting. The whole pipeline has potential applicability in other latent infections as well. ERBB’s role in KSHV lytic is novel and exciting finding. The manuscript is also well written. There are only a few minor revision points to improve the overall manuscript as follow:

**Comment 1.** In this paper, the authors used selection of 29 compounds for their initial screening, which target around half of the human kinome. They should briefly describe the reasoning or the computational set up for this selection.

**Response:** The Kinase Inhibitor Regularization (KiR) approach is based on the fact that all kinase inhibitors (KIs) inhibit multiple kinases but the breadth and potency of each KI for the targeted kinases provides a unique profile. In this approach, an "optimal set" of kinase inhibitors is computationally derived from a matrix of inhibition profiles of 427 compounds against 371 kinases [35]. Unsupervised hierarchical clustering is applied to split the drugs into distinct clusters based on their inhibitory profile. From each cluster, a final collection of drugs is chosen to ensure coverage of many potential kinases, with built-in redundancy since each kinase's residual activity is reduced by at least 30 percent by at least two drugs from this selected set. This results in the collection of inhibitors used in the screen. **We now describe the computational set up more completely (lines 134-144) and refer the reader to several papers** that describe the rationale and methodology in more detail [30, 31, 82]

**Comment 2.** The authors should also address the suitability of these 29 inhibitors as a training set since they are not selective (collectively targeting 296 kinases).

**Response:** As noted in the above response, the core concept is to identify relevant kinases and inhibitors that could affect specific biological characteristics by using experimental data from a subset of drugs (29 inhibitors), rather than testing all 427 KIs. As a result, the selection process prioritizes comprehensiveness over specificity, with the aim of extracting the maximum information on drug-target interactions from the original matrix. The predictive capabilities of this set of inhibitors have been assessed and validated in previous studies. For instance, a regression model built using this representative set was able to (1) identify ‘Fyn” kinase as a mediator of cell migration in mesenchymal cancer cells [27], (2) identify PP121 and SC-1 as inhibitor of castration-resistance prostate cancer cell growth, [82] **,** and (3) identify a set of ‘key’ kinases and inhibitors that could suppress SARS-CoV-2 spike protein-mediated cytokine release in macrophages [30]. **The revised manuscript discusses these points and provides additional data showing the activities of the KIs in inhibiting the 13 kinases identified in the screen as well as the ERBB and MKNK family member (new S3 and S5 Table)**.

**Comment 3.** How selective are the top inhibitors in targeting each of the 13 kinases vs other non-kinase cellular functions? It seems that around 50% of the kinases listed in table s3 are validated.

**Response:** Although our kinase inhibitor profiling methods were designed to target kinases, it is possible that some of the kinase inhibitors also target non-kinase proteins. Nonetheless, the fact that ~50% of the kinases predicted by the screen were confirmed by complementary genetic (siRNA) experiments, along with the demonstrated success of this method in multiple systems [27, 30, 82] suggests that the kinase inhibitory effects of the drugs are their key activities.

**Comment 4**. The authors put forward this model of ERBB2 switching interaction partners from ERBB1 during latency and ERBB3 in lytic, but there is no direct evidence showing of these heterodimerizations and switching.

**Response:** We tried several times, unsuccessfully, to use immunoprecipitation assays to directly observe heterodimers of ERBB2 with the other EBBBs. Among the complicating issues is the very low level of expression of ERBB3 and ERBB4. We were able to detect ERBB3 and ERBB4 following induction of lytic replication by immunoblots (see new **Fig 4A**), but the levels are considerably lower than found in two well characterized cancer cell lines (MCF-7 and BT474). We also attempted to detect phosphorylated ERBB3 and ERBB4 using the reverse phase protein assay described in the manuscript, but the data quality was poor, likely due to the low amounts of the proteins. Although we do not have biochemical evidence for ERBB2 dimers (now noted as a future direction, lines 507-508), we have performed additional experiments that provide support for changes in ERBB-dependent signaling that we have incorporated into our revised model (see responses to comment 5 and modified Fig. 9).

**Comment 5**. The paper should also show interference of ERBB2 heterodimerizations leads to lytic reactivation or lytic inhibition (ie. Latency) to further support the model presented in the manuscript.

**Response: We performed several new experiments** to address this issue.

**ERBB2 likely acts as a heterodimer rather than a homodimer.** We found that the total amounts of ERBB2 (**new Fig 4A**) and phospho-ERBB2 (**new Fig 4B and C**) are similar in latent and lytic conditions. The presence of phosphorylation at several sites indicates that ERBB2 is active in both conditions. The observation that ERBB2 levels are relatively low suggests that ERBB2 is engaged with other ERBBs in heterodimers rather than homodimers, which are thought to form only when the amounts of ERBB2 are high, as in BT474s [111]. The finding that ERBB2 knockdown inhibits reactivation and reduces ERBB1 phosphorylation during latency, but not reactivation (Fig. 6B) suggests that ERBB2 is engaging different partners during these different phases in the viral life cycle.

**ERBB1:ERBB2 heterodimers support latency.** We have now evaluated the effects of tucatinib, an inhibitor of ERBB2. Tucatinib inhibits ERBB2 specifically at low concentrations, but at higher concentrations it also inhibits ERBB1 [42]. Consistent with ERBB2 playing a role in reactivation, low concentrations of the drug (31nM) reduced KSHV reactivation slightly (**new Fig 4E**). At 125nM, levels of reactivation returned to control levels, possibly due to tucatinib also inhibiting the pro-latency effects of ERBB1 at this higher concentration**.**

**ERBB2 phosphorylates ERBB1 in latently infected cells.** We had shown that knocking down ERBB2 reduces phospho-ERBB1 levels during latency, but did not reduce it under lytic conditions (Fig 6B). We now find that low dose tucatinib also slightly, but significantly, inhibited ERBB1 phosphorylation under latent conditions but had no effect on phospho-ERBB1 levels under lytic conditions (Rebuttal Fig 1). (For unknown reasons, the phospho-ERBB1 levels were unusually low in the tucatinib experiment compared to the data in Fig 6B.) Together, these data suggest that ERBB2 activates ERBB1 during latency, but is needed to signal with other partners to enable efficient reactivation.

**Rebuttal Fig 1.** ERBB2 kinase inhibitor reduces ERBB1 phosphorylation in latently infected cells. RPPA was performed as described in the paper.

**Role of ERBB2:ERBB3 heterodimers in reactivation.** It is known that ERBB2 preferentially binds to ERBB3 [39], suggesting that when ERBB3 expression increases during reactivation (Rebuttal Fig 1), ERBB2 is likely to interact with it. The low amount of ERBB3 limited our ability to detect phospho-ERBB3 using our reverse phase assay. However, we did detect a slight increase in ERBB3 phosphorylation following lytic replication induction and a slight decrease after lytic induction plus tucatinib treatment (Rebuttal Fig 2). Though not significant, these trends are consistent with our model (that ERBB2 partners with ERBB3 during KSHV reactivation).

**Rebuttal Fig 2.** Phospho-ERBB3 levels are modestly increased following induced lytic replication but reduced if combined with the ERBB2 inhibitor tucatinib. RPPA was performed as described in the paper.

**ERBB2 and ERBB3 regulate downstream transcription factor phosphorylation.** We showed in the manuscript that knocking down ERBB2 and ERBB3 results in quantitatively similar inhibition of reactivation as measured by mCherry-nls protein expression (Fig 4) as well as viral lytic cycle RNAs (Fig 5), consistent with them acting together. We have now tested the effects ERBB3 on downstream factors. These experiments show that knocking down ERBB3 reduces activation of CREB1, STAT1, and STAT3 (**new Fig S7**), just like knocking down ERBB2 (Fig 8), consistent with signaling from the ERBB2:ERBB3 heterodimer during reactivation. ERBB4 knock down did not significantly impact the phosphorylation of these transcription factors, possibly because its expression is very low (new Fig 4A) or because it has an alternative mechanism to promote reactivation.

Thus, although we were not able to demonstrate EBBB2 heterodimer switching directly, our provide consistent support our hypothesis that ERBB2 acts with ERBB1 during latency and with ERBB3 and possibly ERBB4 during reactivation. **We have revised our discussion of these points in the results (pp. 6 and 7) and discussion (pp. 10-12) sections of the manuscript, added data to the manuscript (Figs 4 and S7), and modified our working model (Fig 9).**

**Comment 6**. How do the authors ascertain ERBB2:3 vs ERBB2:4 induces CREB1 phosphorylation and lytic reactivation (Fig 9) since ERBB4 is one of the strongest pro-lytic factors found from their screening and validation (Fig 3)? Exogenous expression of ERBBs or other more appropriate cell lines can be used if endogenous expression is low.

Does ligand binding affect the heterodimerization switching? Or if any other conditions induce the switching? It would be nice to link it to KSHV pathogenesis.

**Response:** As noted in response to comment 5, we tested the role of ERBB3 and ERBB4 in the regulation of CREB1 as well as two other transcription factors, STAT1 and STAT3. The results show that ERBB3 regulates STAT1 and likely CREB1 phosphorylation (**new S7 Fig**). ERBB4 knockdown did not significantly impact the phosphorylation of these transcription factors, possibly because its expression is very low or because it has an alternative mechanism to promote reactivation. Thus, these data suggest that ERBB2 and ERBB3 signaling activates CREB1 but do not clarify the role of ERBB4.

Since ERBB3 is catalytically inactive and not part of the 371 kinase panels, it was not possible to determine the role of ERBB3 using the KIR models. Although ERBB4 was identified as a top hit in the screen, when we knocked down each of the ERBBs independently, ERBB4 was consistently less potent than ERBB2 and ERBB3 (Figs 4 and 5).

While we agree that determining the roles of ERBB ligands in the proposed switch and in KSHV pathogenesis will ultimately be necessary to completely understand how the EBBB family acts during KSHV infection, we feel that those studies are beyond the scope of this report (now noted as a future direction, line 508).

**We now discuss these issues in the results and discussion.**

Part I - Summary
Please use this section to discuss strengths/weaknesses of study, novelty/significance, general execution and scholarship.

Reviewer #1: Olson et al., employed a polypharmacology-based kinome screen to identify specific kinases that regulate KSHV reactivation. Results identified several kinases that enhance reactivation, whilst other promote the maintenance of latency. Further experiments focus on the potential mechanism of ERBB2 signaling.

This is a potentially interesting study examining the essential role of host cell kinases in the KSHV life cycle. It merits publication in the future, however, overall conclusions are unsubstantiated and require further validation and evidence, especially on mechanistic signalling claims.

Reviewer #2: (No Response)

Reviewer #3: In this manuscript, the authors use a polypharmacology-based kinome screen to search for kinases that support or suppress KSHV lytic reactivation, once triggered by inducible RTA expression and sodium butyrate (NaB) treatment. They engineer an elegant reporter system in iSLK cells, in which lytic gene expression is readout by a PrPAN driven mCherry reporter. This system is then used for a polypharmacology based kinome screen for enhancers or repressors of NaB/Dox induced reactivation. The idea is that kinase inhibitors have known sets of on/off target activity, and computational approaches can then be used to triangulate the relevant host cell kinase targets. The screen identified a number of host cell kinases, which were validated by chemical and siRNA approaches. Effects of TEK family kinase inhibition were noted, but the authors mostly focus on the interesting findings that ERBB2/3/4 inhibition impairs lytic replication, whereas ERBB1 was found to have a pro-latency role. Kinase inhibition alone was not sufficient to induce lytic replication, but functioned in the context of cells stimulated by Dox/NaB. Phosphoproteomic studies were used to characterize effects of iSLK lytic reactivation -/+ ERBB2 or downstream kinase blockade. Overall, this is an interesting study, which serves both as a proof-of-concept for the chemical screen approach in KSHV reactivation, which identified pro-latency/reactivation roles of ERBB kinases. The manuscript is well written and the figures are concisely and clearly presented. I would suggest addressing the following points prior to publication.

Reviewer #4: In the work described in this manuscript, Olson et al attempted to identify cellular kinase signaling pathways that regulate KSHV reactivation. They first engineered a reporter virus for easy cell-based assay; they next screened a small set of kinase inhibitors and then a larger set of inhibitors. Based on vaguely described Kinase Inhibitor Regularization (KiR) analyses, the authors selected 13 kinases for further analyses. SiRNA knock-down of the ERBB4(HER4) and MKNK2 (MNK2) noticeably reduced KSHV reactivation while knock-down of DSTYK (RIPK5), ITK, and TEC had moderate effects. The authors focused mostly on ERBB4 and MKNK2, and then examined the homologous kinases using similar approaches. Surprisingly, they found some of the homologues had the opposite effects. For example, they showed that ERBB2, ERBB3, ERBB4, and MKNK2 had pro-lytic/viral effects while ERBB1 and MKNK1 had anti-viral effects. They proposed that ERBB1 and ERBB2 form dimers in cells and the heterodimers between ERBB1 and other partners regulate KSHV reactivation. They also analyzed the downstream kinases and potent protein substrates. They provided evidence that CREB1 and JAKs might be involved. Overall, this chemical biology approach is novel and the results are interesting. However, the methodology was not described in enough detail. I had difficulty understanding how the top hit kinases were generated by the model, specifically how the experimental data were fitted with the known inhibitors profiles to produce the top hits. Furthermore, some of the experimental data were mostly correlative, inconclusive, and sometimes conflicted with each other.

Please see below for responses to the specific issues raised in these summary comments.

Summary.

**Part II – Major Issues: Key Experiments Required for Acceptance**

Please use this section to detail the key new experiments or modifications of existing experiments that should be absolutely required to validate study conclusions.

Generally, there should be no more than 3 such required experiments or major modifications for a "Major Revision" recommendation. If more than 3 experiments are necessary to validate the study conclusions, then you are encouraged to recommend "Reject".

Reviewer #1:

**Comment 7**: Throughout the paper is it pro-latent or anti-viral? More analysis is required to evidence this claim.

**Response: We have modified the text** to clarify that we are measuring the impact of drugs and kinases on the promotion or inhibition of reactivation and use “pro-latent” to mean inhibition of reactivation (lines 200-202). We do not use “anti-viral.”

**Comment 8**: Line 137: More details would be helpful on how the computer selection occurred. Are the kinase selected upstream or downstream in a signalling cascade as any effects could be non-specific. How were these concentrations chosen? For example, all the concs used for lestaurtinib are above the IC50 for 3 major targeted kinases (JAK2, FLT3 and TrkA inhibitor (IC50 values are 0.9, 3 and < 25 nM, respectively). Considering this information, it is highly unusual no induction of apoptosis is seen. It needs to be shown that the inhibitors used are functioning, although I acknowledge this may not be possible for all the inhibitors used in the screen, confirming that the inhibitors are working at the concentrations used should be done for any inhibitors that are of downstream importance.

**Response:** Please see responses to comments 1, 2 and 3 above.

Regarding the concentration and toxicity issues - the initial 29 KIs were tested at a range of concentrations (31.5 nM, 125nM, 500nM, and 2uM). The 500 nM concentration, at which we observed effects on reactivation with little effect on cell viability, was used in the analyses. Even so, we did observe that seven of the 29 KIs resulted in reduced cell viability and these data were excluded from the analyses. The second round of KIs testing included 8 μM and at this higher concentration, we observed more toxicity (revised Fig 2D). Note that KSHV expresses proteins that block apoptosis, and thus the toxicity of these drugs may vary compared to other cell types [112, 113]. Further, the IC50 noted for lestaurtinib is based on an *in vitro* kinase activity profiling. The cellular IC50 is usually higher than in vitro-determined kinase activity inhibition. Thus, we think our measurements of cell viability in the presence and absence of KIs are reasonable empiric measurements and allow analyses of the effects on reactivation in cells with similar viability. Finally, all 427 compounds were quality controlled at NCATS and were functionally tested in kinome profiling assays. The kinase inhibitors used for the initial screen were from the same batch. Instead of validating that the kinase inhibitors are functional, we focused on the more specific approach of validating hits from the screen by siRNA depletion experiments.

**Comment 9**: Line 213: The data is not strong enough to make this statement.

As noted in response to comment 5 above, **we have added new data and changed the text supporting this statement on pp. 6 and 7.**

**Comment 10**: Line 276: Authors claim a downstream target of PKC is phosphorylated but earlier show PKC itself is not phosphorylated/activated (line 243).

**Response:** The antibody used to detect PKC phosphorylation detects multiple isoforms, which can phosphorylate MARCKS at different rates [48] . Additionally, MARCKS can be phosphorylated at Ser152/156 by ROCK, suggesting that MARCKS can be regulated independent of PKC [49, 114]. W**e now discuss these points in the text (lines 287-289, 321)**.

**Comment 11**: Fig 2B: where are the dose response curves for the other kinases? Only 2 are shown - Some missing error bars.

**Response: We have** **added the dose response data** for all of the 19 (out of 29 total) non-toxic kinase inhibitors with consistent dose responses that were used in the KiR analysis (**new S1 Table**). The error bars in figure 2B are very small, so minor adjustments to the error bar coloring and display have been made to make them more visible.

**Comment 12**: FigS2: error bars. Lacking of detail in legend. How many cells were used to calculate this.

**Response:** Under these conditions (no treatment with DOX and NaB), the 29 kinase inhibitors were all tested once and a subset, which showed some toxicity or high variability, were tested a second time. Therefore, the data in Figure S2 do not have error bars and represent the average of 3 technical replicates from a single experiment or the average from two separate experiments in the case of KIs that were repeated. **We have revised the Fig S2 legend** to explain that 0.02% corresponds to only ~2 mCherry positive cells.

**Comment 13**: Fig3: The x axis for KD is unclear, are the authors using KDs that haven’t worked in this experiment? Large error bars. WB would be good to confirm KD.

**Response: We have clarified in the legend of Fig 3** that “% KD” below the X-axis refers to the average knock down as measured by RT-qPCR. The possibly confusing designation “ND” under ITK is now explained in the Fig 3 legend and we point out that the % KD for each individual biological replicate is shown in Fig S3 and S4. The reactivation data in Fig 3 incorporate all the replicates, including those experiments in which the knock down did not work as well. We think that immunoblot data would not add much to the RT-qPCR data to enhance the key points of the paper.

**Comment 14**: FigS3: Confusing : what it is normalised to? as siCtrl are defined as 100% but often come up below 1.0 on the graph. There appears to be strong changes between ctrl and sictrl levels. Large variation in KDs, e.g. is RIPK7. ERBB4 KD data is missing compared to Fig3B. Additionally, functional KD of downstream signalling pathways should be given i.e. ERBB4 KD should lead to changes in PI3K, ERK and STAT signalling pathways. The KD of these genes alone gives no indication of the correct downstream effect. Additionally, FigS5: Why does ERBB2 KD not decrease p-MARCKS under normal conditions?

##### **Response:** The experiments in Figs S3 and S4 included un-transfected as well as non-targeting siRNA transfected controls. Thus, the difference between 1 on the Y axis and the 100% labels above “siCtrl” reflects the relatively small effects of the transfection protocol itself. We emphasize the efficiency of the gene-specific knockdown by showing it as a % of the siCtrl. **We now explain the transfection controls used to normalize the data in the legends to Figs S3 and S4.**

The ‘missing’ ERRB4 knockdown data are shown in Fig 4 and S4, along with that for the other ERBBs and MKNKs as **we note in the legends to Figs 3 and S3. We also** **clarify that the ERBB4 data shown in Fig 3 are shown again in Fig. 4 for clarity.**

We present the data on downstream signaling later in the paper (see Fig S5, 6-8). We discuss ERBB4 specifically above (response to comment 5).

Although there are reports that ERBBs might be upstream of ROCK (which phosphorylates MARCKS [49]) basal levels of MARCKS phosphorylation do not depend on ERBB2 in our latently infected cells.

**Comment 15**: Fig4: The *** significance appears less significant than some of the * on the neighbouring graph with no explanation of the calculation. Also Fig 6: are these significant?

**Response:** The difference in the appearance of the significances in Fig 4 stems from the fact that the data in Fig 4A came from three independent experiments while that in Fig 4B came from only two. We have indicated the differences that are significant in Fig. 6. All other comparisons are not significant, as is true in all the figures when conditions are being compared. The statistical calculation methods are explained in the figure legends and methods section.

**Comment 16**: Line 240: It is unclear why there are so many repeats used for his experiment. Additionally, the decrease in p-AKT is not demonstrated to be relevant for the mechanism presented. To prove AKT signalling is needed, AKT should be directly targeted and the same effect should be seen (lytic reactivation, early and late gene and protein expression). Additionally, several AKT inhibitors can be found in table s2 but the predicted effect is very minor, contradicting the mechanism described here.

This comment can also be applied to ERK1/2 and SRC.

**Response:** In one of the initial ERBB2 knock down experiments, the control KSHV reactivation level was unusually low for unknown reasons, so we repeated it 3 extra times.

We agree that the role of phosphorylated AKT is uncertain from our data. However, we do show that it and the other kinases tested (SRC and ERK1/2) are activated during latency and their activation status follows the pattern of ERBB1 (Figs 6 and 7), which is upstream of these kinases. Like ERBB1, AKT phosphorylation is dependent on ERBB2 in latently infected cells. However, in cells containing reactivated virus, ERBB2 no longer plays a significant role in AKT phosphorylation. This supports our working model that in latent cells, ERBB1:ERBB2 heterodimers activate AKT phosphorylation.

The fact that not all KIs with activity against AKT impact reactivation is not a strong argument against it having a role, as these KIs have multiple other targets. Thus, siRNAs experiments are needed to assess the roles of specific kinases, but we feel that additional knock down experiments to trace the signaling intermediate is beyond the scope of this paper. ERBB signaling is exceedingly complex and dissecting these pathways, as well as the roles of ERK1/2 and SRC will require the construction of additional tools and experimentation.

**Comment 17**: Line 268: This conclusion cannot be drawn from the data, it seems more likely that the JAK3 inhibitor VI is having an off-target effect. Additionally, no link between JAK1 and AKT/SRC/ERK1/2 is shown.

**Response:** We agree and **note that JAK3 inhibitor VI has broad kinase inhibitory activity (S7 Table).** To emphasize this point, we now report that this drug inhibits 113 kinases >50% at 500 nM in an added Table S5. To further support this conclusion, none of the individual JAK family protein knock downs affected reactivation in the presence of DOX plus NaB (Fig 7C). We have modified line 313 to avoid suggesting that we have shown a link between JAK1 and AKT/SRC/ERK1/2 as downstream intermediates of ERBB1:ERBB2.

**Comment 18**: Line 288: Why was CREB chosen to be important, compared to the much larger change in STAT?

Additionally, there is not enough evidence linking ERBB2 and CREB. More validation is needed, such as use of inhibitors, D/N mutants etc.

**Response**:

**We have modified the text** to include more discussion of the effects of STATs as well as CREB1 (pp. 8-9). Consistent with our findings (Fig. 8), prior studies have reported roles for CREB1 in KSHV reactivation [18] and replication of other DNA viruses [57-59, 62]. As well, a signaling connection between ERBB2 and CREB1 phosphorylation has been reported in other systems [68, 69].

Direct and indirect regulation has been documented for the ERBBs upstream of STATs as well. Specifically, ERBB2 has been linked to STAT1 Tyr701 phosphorylation [70] and STAT3 Tyr705 can be phosphorylated by ERBB1 *in vitro* [71] and in cancer cells [72, 73], or occur in response to ERBB2 overexpression [74].

Our knockdown data suggest that ERBB2 (Fig. 8) and now ERBB3 (**new Fig S7**) contribute to phosphorylation of these factors in reactivating conditions. We do agree that our data do not reveal the intermediate factor(s) that link ERBB2 to CREB1 or STATs.

**Comment 19**: Fig 9: Overall proposed schematic. This is rather over-interpreted from the data provided. The authors provide no evidence to show an ERBB1:ERBB2 interaction and that this interaction is disrupted in early lytic replication.

**Response**: Please see our responses to comments 4-6. In brief, we have provided new supporting data and have modified Fig 9 to emphasize that, while we have evidence for the role of ERBBs in signaling, we have not demonstrated the formation of the postulated heterodimers.

Reviewer #2: (No Response)

Reviewer #3

**Comment 20**: Initial screen could be confounded by effects on dox-induced RTA. Figure 3 should include a western blot to indicate effects on chemicals on RTA expression at an early timepoint of Dox induction (prior to downstream effects on RTA autoinduction via the lytic cycle). At a minimum, it would be important to show that the ERRB inhibitors do not simply function by perturbing the level of RTA driven by DOX the inducible system.

**Response**: **We have added data showing *RTA* expression is not significantly altered by ERBB knock down** under uninduced or induced conditions (**new Fig 5A and B**). While it is possible that some drugs used in the initial screen may have affected *RTA* expression, these new data argue that the effects of ERBB knockdown on reactivation are independent of changes in *RTA* expression.

**Comment 21:** Would show levels of ERBB1-4 by qPCR or western blot for each knockdown, to also show the extent to which a given siRNA affects other closely related ERBB family members in Fig 4/S4.

**Response**: We think the Reviewer may have overlooked Fig S4, in which we demonstrated good specificity of each siRNA for the corresponding ERBB by RT-qPCR.

**Comment 22:** Given the model, would present phosphokinase analysis for the other ERBB kinases as well, ERBB2-4. Data is presented only for ERBB1, but the data appears to be available for all ERBB kinases. Likewise, since a global phosphopeptide analysis was done for Fig 6-7, it would be nice if a volcano plot or some similar systematic analysis could be presented, to highlight the extent to which effects on ERBB phosphorylation and pathways are specific. For instance, based on the data we are shown, it is difficult to know whether many kinases are similarly affected by lytic reactivation, or whether the effects on ERBB are specific, which would nicely support the authors model.

**Response**: We have now analyzed total ERBB1-4 levels by immunoblot, phospo-ERBB2 by immunoblot assay and performed the reverse phase protein array (RPPA) to investigate phospho-ERBB2-4 levels. The phospho-ERBB2 data (**new Figs 4B and C**) demonstrate that ERBB2 is phosphorylated in both latent and lytic inducing conditions, supporting the conclusion that it is in active complexes. However, total ERBB3 and ERBB4 levels were very low even under reactivation conditions (**new Fig 4A**). We detected a slight increase in phospho-ERBB3 upon reactivation (Rebuttal Fig 2) but these levels were very low for this assay and we did not detect phospho-ERBB4 (data not shown).

We analyzed only 14 proteins in our reverse phase protein arrays (RPPA). Thus, we do not have a “global” phosphopeptide analysis to present. All the RPPA data we have are represented in Figs 6, 7, 8, S5 and S7.

**Comment 23:** Since this is important to the model, please provide more information about CREB1 Ser133 phosphorylation. Is this the key site that indicates CREB1 activation status? Were other CREB1 phosphorylation sites tested, or was this the only one on the array? Would likewise provide this information for STAT1 and STAT3.

**Response**: CREB1 Ser133 is a key phosphorylation site targeted by several kinases that enables the recruitment of coactivators, CBP and p300, as revealed in studies using S133A mutants [50, 52, 53, 55]. Published work demonstrates a role for CREB1 in promoting KSHV lytic gene expression dependent on MSK1/2, which phosphorylate CREB1 at Ser133 [18, 115]. No other CREB1 phosphorylation sites were assayed in our studies.

Similarly, the STAT1 and STAT3 phosphorylation sites selected are key sites that indicate activation [51, 54, 56]. KSHV vIL-6, an early lytic gene, mediates STAT3 Tyr705 phosphorylation [65, 116]. ERBB2 can phoshorylate STAT1 at Tyr701 [70] and STAT3 Tyr705 ([74].

**We have now referenced the importance of these sites** **in lines 335 and 337-341.**

**Comment 24:** To further support the model in Fig 9, would show that CREB1 knockdown reduces lytic gene expression in their model system. Why might CREB1 act apparently downstream of RTA? Would clarify in the model that CREB1 acts downstream of or at least in parallel with RTA (at present, the model doesn’t capture the fact that Dox/NaB were upstream activators).

**Response**: Previous analyses of initial (not reactivated) infection revealed that CREB1 was upstream of RTA expression (Cheng et al.). RTA and CREB1 share coactivators CBP and p300, but bind different domains of CBP [117], suggesting they could be acting together. In our system, we induce *RTA* expression using DOX, and thus bypass any prior requirement for CREB1 to activate RTA expression. We do not know whether CREB1 phosphorylation is a result of RTA expression or is an effect of NaB, as we know explain in the text (**lines 461-2**).

Reviewer #4:

**Comment 25:** 1. The Kinase Inhibitor Regularization (KiR) platform seems to make sense, but the method was not described in enough detail. Despite reading the manuscript a few times, I was still unable to figure out how exactly the top hits were generated, and therefore had a number of questions. For example, both top hits KI lestaurtinib and K252a are broad spectrum kinase inhibitors, what are the key experimental screening results that could single out ERBB4 and MKNK2 among their homologues? How many of the top hit kinase inhibitors could distinguish ERBB and MKNK homologues, or showed different inhibition profiles against ERBB homologues? Are there any more specific ERBB and MKNK inhibitors that can be used to lend more supportive evidence?

**Response**: Please see our responses to comments 1-3 for more details about the KiR screen methodology. For the predicted and tested kinase inhibitors, **we now provide tables that show which of these drugs inhibit the specific kinases on our list, including the ERBB and MKNK family members (Tables S3 and S5) and show data for another ERBB KI, tucatinib** (see response to comment 5, new Fig 4E). However, because the drugs have overlapping inhibitory profiles within closely related kinase families, the ability of the screen to resolve which single kinase is responsible for the phenotype is imperfect and is the reason we tested the predictions by siRNA depletion.

**Comment 26**: If indeed ERBB and MKNK families of kinases are important for KSHV reactivation, can the KiR model predict more specific inhibitors to these kinases? If yes, can those inhibitors be used as did the JAK inhibitors in this study? Because several downstream kinases are involved in the ERBBs signaling, can the KiR model predict more specific inhibitors for each downstream kinase? Use of those kinase inhibitors individually or in combinations to inhibit single or multiple pathways would provide a good validation and pinpoint which downstream kinase(s) is/are more critical.

**Response**: The KiR screen can predict many kinase inhibitors that might influence KSHV reactivation, but these are not specific (see changes to new Table S3). For this reason, we performed siRNA knock downs to validate the predicted kinases. Similar to our JAK kinase inhibitor studies, there are KIs with more specificity to the ERBBs. We have not tested two of these, tucatinib and erlotinib. Tucatinib inhibits ERBB2 at low concentrations while at high concentrations it also inhibits ERBB1. This drug modestly reduced KSHV reactivation at 31nM, but this reduction was eliminated at 125nM (new Fig 4E). Erlotinib is an inhibitor of ERBB1 catalytic activity and consistent with original Fig. 4A (new Fig 4D), this drug had no effect on KSHV reactivation (Rebuttal Fig 3). For pathway analyses, we think that probing of several downstream kinases (Fig 6, 7, S5, and 8), represents a start to dissecting the relevant ERBB2 signaling intermediates, but agree that more mechanistic work will need to be done to fully understand the pathway.

**Rebuttal Fig 3.** KSHV reactivation following erlotinib treatment and induction of lytic replication. KSHV reactivation assay was performed as described in the paper.

**Comment 27**: 3. For validation of the ERBB and MKNK kinases, SiRNA down experiments seem not enough. Constitutive active, kinase-dead, and dominant negative mutants should be used too.

We agree that these are good experiments for future testing of our model. This screening approach has proven to be predictive in several settings (ref 27, 30 and PNAS 2021). Therefore, our kinome screen and validation by specific siRNA depletion experiments reveal valuable new clues into the regulation of KSHV reactivation. Our evaluation of the impact of these kinases on KSHV lytic gene expression and downstream signaling (Figs 5-8, S5, and new data in Fig S7) provides a foundation for future studies that should include the use of kinase mutants, as the reviewer suggests.

**Comment 28:** 4. The heterodimer model of ERBBs seems appealing but the model was not validated by any experiments.

**Response**: Please see responses to comments 4-6 and our revised working model (Fig. 9).

**Comment 29**: 5. Do ERBBs directly phosphorylate CREB1 at Ser133? If not, which downstream kinase would be responsible? Any data to support that kinase is involved?

**Response**: Please see responses to comments 18 and 23. It is unknown if in our system ERBBs phosphorylate CREB1 at Ser133 directly or indirectly. One possible mechanism is that ERBB-dependent activation of p38 MAPK (Plotnikov 2011, Mol Cell Research. Review) triggers MSK1/2 activation that then mediates CREB1 Ser133 phosphorylation [115]. In KSHV infected cells, MSK1/2 is known to phosphorylate CREB1 on Ser133 [18].

**Comment 30**: 6. Because ERBBs are EGF receptors, it would be interesting to examine whether EGF signaling can modulate KSHV reactivation.

**Response**: We agree that the impact of ERBB family ligands is an interesting topic, as we now comment on in the Discussion, but we feel that those studies are beyond the scope of this report.

**Part III – Minor Issues: Editorial and Data Presentation Modifications**

Please use this section for editorial suggestions as well as relatively minor modifications of existing data that would enhance clarity.

**Comment 31:** Reviewer #1: Statistical analysis is unclear with no method stated.

**Response**: **We have addressed this by adding the statistical methods to the figure legends.**

Reviewer #2: (No Response)

**Comment 32:** Reviewer #3: Typo in line 103: known to activat

**Response**: **We corrected this typo**.

**Comment 33:** Fig 2. Would enlarge * as they are quite small when viewed without enlarging the figure.

**Response**: **We have made the suggested changes in the sizes of the asterisk symbol throughout the manuscript.**

**Comment 34:** Line 145. Would better describe what is meant by 7 compounds being removed due to toxicity.

**Response**: **We clarified the definition of toxicity** in lines 154-156 and made similar changes in the legends of Figs 2 and S2.

**Comment 35:** Line 277, would clarify “ERBB2 knock down lessened the induction but not to a statistically significant extent”. I think this is a typo, and rather than induction, phosphorylation is meant here.

**Response**: **We changed “induction” to “phosphorylation.”**

**Comment 36:** Given concerns that there is very low expression of ITK, and therefore that 5-fold induction may still be very low levels of ITK, the question is raised of whether the single siRNAs used may have off-target effects. This could be addressed by demonstrating blotting ITK knockdown samples for both ITK and TEC.

**Response**: As noted in the methods and in original Table S6 (new Table S9), we used pools of 4 siRNA, not single siRNAs, targeting each gene. Although we cannot rule out off-target effects in our system, these siRNA pools were purchased from Dharmacon with high performance and specificity assurances. Also, ERBB and MKNK siRNA pools demonstrated good specificity (original Fig S4B and C; new Figs S3B and S4B). Nonetheless, we agree that further validation with immunoblots and/or RT-qPCR assays will be needed in future evaluation on the roles of ITK and TEC in reactivation.

**Comment 37:** Would clarify in the figure legends whether technical or biological replicates were used. Would add to the fig legend what was used as the load control for the qPCR analysis.

**Response**: We now provide the information about the number of technical and biological replicates in the Materials and Methods, and report the control for the RT qPCR analyses in the Fig legends.

**Comment 38**: Would add to fig 6 legend how protein levels were normalized, given that this is the Y axis label.

**Response**: We have clarified the protein levels were normalized to the un-transfected control samples, as we now note in legends for new Figs 6B/C, 7A, S5A/B, 8, S7.

**Comment 39**: Fig 7C. would add replicates to the tofacitinib experiments so that lack of significance can be properly assigned, given also the very large variance between the 2 replicates.

**Response**: We feel there is little to be gained by additional replicates of this experiment because we performed the more precise JAK family gene-specific knock downs (Fig 7C).

**Comment 40**: Would clarify an apparent paradox. It is stated in line 380 that during lytic replication ERBB2 does not appear to activate these signaling intermediates. And yet, ERBB2 was required to fully activate CREB1 (Fig 8).

**Response**: As discussed in responses to comments 18 and 23, our observation is that ERBB2 is required for full CREB1 activation. We also show that ERBB2 does not greatly impact the intermediates SRC and ERK1/2 upon lytic induction, which suggests that ERBB2 effect on CREB1 is mediated by another as yet unidentified factor but could include p38 MAPK and MSK1/2.

**Comment 41:** Reviewer #4: Although different concentrations of the inhibitors were used in the screening, it appeared that only the data from a single concentration (0.5uM) were used for the analysis (Table S2). Please explain.

**Response**: Although several concentrations were experimentally tested, the initial KiR modeling utilizes a single concentration for all 29 drugs. However, the final optimized KiR model was utilized to predict responses to 427 kinase inhibitors at three different concentrations (50nM, 500nM, and 2000nM) (new Table S4). We selected 500nM as the optimal concentration for the KiR model building because at this concentration, we observed reactivation phenotypes for many of the drugs, and only a few reduced the cell confluence by > 30% compared to the DMSO control condition (our measurement of cell viability).

**Comment 42:** More detailed legends or descriptions are needed for those tables, so readers can understand how the data were generated. The “Predicted KI responses at 0.5 uM” in the Table S2 and “Average Coefficient” in the Table S3 were not defined or explained. They are not self-explanatory to me.

**Response**: **We have added explanations as requested**. In original Table S2 (new Table S3), the “predicted KI response” is the expected level of KSHV reactivation when cells are incubated with the drug at that concentration. Column A denotes the rank of kinase inhibitors, Column B denotes the name of kinase inhibitor, and Column C denotes the KiR-predicted response at the indicated dose. We now show the predicted responses normalized to DMSO control (set to 100%).

The original Table S3 (new Table S4) shows KiR-predicted, unranked, list of ‘informative’ kinases. We removed the numbers in column A. The top 14 kinases predicted by KiR are listed in the Table. We also removed the model “average co-efficient” column as this value is not biologically meaningful

**Additional References**

111.	Peckys, D.B., et al., *Visualisation of HER2 homodimers in single cells from HER2 overexpressing primary formalin fixed paraffin embedded tumour tissue.* Mol Med, 2019. **25**(1): p. 42.

112.	Wang, H.W., et al., *Characterization of an anti-apoptotic glycoprotein encoded by Kaposi's sarcoma-associated herpesvirus which resembles a spliced variant of human survivin.* EMBO J, 2002. **21**(11): p. 2602-15.

113.	Bellows, D.S., et al., *Antiapoptotic herpesvirus Bcl-2 homologs escape caspase-mediated conversion to proapoptotic proteins.* J Virol, 2000. **74**(11): p. 5024-31.

114.	Brudvig, J.J. and J.M. Weimer, *X MARCKS the spot: myristoylated alanine-rich C kinase substrate in neuronal function and disease.* Front Cell Neurosci, 2015. **9**: p. 407.

115.	Arthur, J.S., *MSK activation and physiological roles.* Front Biosci, 2008. **13**: p. 5866-79.

116.	Chen, D., et al., *Determinants of secretion and intracellular localization of human herpesvirus 8 interleukin-6.* J Virol, 2009. **83**(13): p. 6874-82.

117.	Gwack, Y., et al., *CREB-binding protein and histone deacetylase regulate the transcriptional activity of Kaposi's sarcoma-associated herpesvirus open reading frame 50.* J Virol, 2001. **75**(4): p. 1909-17.
